# Coexistence of paternally-inherited *ABCC8* mutation and mosaic paternal uniparental disomy 11p hyperinsulinism

**DOI:** 10.1186/s13633-020-00083-5

**Published:** 2020-07-10

**Authors:** Joanna Yuet-ling Tung, Sophie Hon Yu Lai, Sandy Leung Kuen Au, Kit San Yeung, Anita Sik Yau Kan, Florence Loong, Diva D. DeLeón, Jennifer M. Kalish, Arupa Ganguly, Brian Hon Yin Chung, Kelvin Yuen Kwong Chan

**Affiliations:** 1 Department of Paediatrics, Hong Kong Children’s Hospital, Kowloon, Hong Kong; 2 Department of Paediatrics and Adolescent Medicine, Queen Mary Hospital, The University of Hong Kong, 102 Pokfulam Road, Pokfulam, Hong Kong; 3 Prenatal Diagnostic Laboratory, Department of Obstetrics and Gynaecology, Tsan Yuk Hospital, Room 314, 3/F, 30 Hospital Road, Sai Ying Pun, Hong Kong; 4 Department of Pathology, Queen Mary Hospital, The University of Hong Kong, Pokfulam, Hong Kong; 5 Division of Endocrinology and Diabetes, The Children’s Hospital of Philadelphia, Philadelphia, USA; 6 Department of Pediatrics, Perelman School of Medicine at the University of Pennsylvania, Philadelphia, PA USA; 7 Division of Human Genetics, The Children’s Hospital of Philadelphia, Philadelphia, PA USA; 8 Department of Genetics, Perelman School of Medicine at the University of Pennsylvania, Philadelphia, PA USA

**Keywords:** Congenital hyperinsulinism, Hyperinsulinism, Beckwith-Wiedemann syndrome, UPD11

## Abstract

**Background:**

Beckwith–Wiedemann syndrome (BWS) is an overgrowth syndrome with variable clinical phenotype and complex molecular aetiology. It is mainly caused by dysregulation of the chromosome 11p15 imprinted region, which results in overgrowth in multiple tissues, often in a mosaic manner.

**Case presentation:**

A large-for-gestational-age infant without any other somatic features of BWS presented with medically refractory hyperinsulinism (HI) requiring 80% pancreatectomy. Next generation sequencing with congenital HI sequencing panel identified a pathogenic *ABCC8*:c.1792C > T (p.Arg598Ter) variant of paternal origin, suggestive of focal HI. However, pancreatic histology revealed atypical findings of coalescing nests and trabeculae of adenomatosis scattered with islets with isolated enlarged, hyperchromatic nuclei scattered throughout the pancreas. Methylation analysis, SNP-based chromosomal microarray and short tandem repeat markers analysis revealed mosaic segmental paternal uniparental disomy (UPD) 11p15.5-p15.1 in the pancreatic tissue, but not the peripheral blood, suggestive of BWS/BW-spectrum HI.

**Conclusions:**

This case highlights the importance of integrating the clinical presentation and subsequent clinical course, together with radiological, genetic and histological findings in the definitive diagnosis of this rare yet clinically important entity. In addition, this is the first report that demonstrated the level of paternal inherited c.1792 T pathogenic variant in the pancreatic tissue being directly correlated to the mosaic level of pUPD.

## Background

Congenital hyperinsulinism (HI) is the most common cause of persistent hypoglycaemia in infants. It is characterized by dysregulated insulin secretion from pancreatic β-cells and is a group of heterogeneous conditions that vary in terms of clinical severity, histopathology and molecular aetiology. Inactivating mutations of the *ABCC8* and *KCNJ11* genes, which are located on 11p15.1 and encode the SUR1 and Kir6.2 subunits of the pancreatic β-cell ATP-sensitive potassium channel (K_ATP_ channel) respectively, are the most common genetic aetiology of HI [1].

There are two major histological subtypes — diffuse and focal HI. The two have distinct molecular aetiology and response to medical treatment. Rarely, some patients have atypical histology that could not be easily classified into either focal or diffuse forms [2]. They have enlargement of β-cell nuclei that is distinct from diffuse HI in several discrete regions of the pancreas, which suggests the possibility of mosaicism [3].

Beckwith–Wiedemann syndrome (BWS) is an overgrowth syndrome with variable clinical phenotype and complex molecular aetiology. It is mainly caused by the dysregulation of the chromosome 11p15 imprinted region, which results in overgrowth in multiple tissues, often in a mosaic manner [4]. While only a small proportion of HI are associated with BWS, transient HI occurs in up to 50% of BWS neonates, and 5% have persistent HI requiring medical and/or surgical management [5, 6]. The exact mechanism of HI in patients with BWS has remained unclear. In a cohort of children with HI and BWS, it was demonstrated that most did not have a concomitant K_ATP_ defect, however they did have pancreatic lesions significantly larger than those seen in cases of focal HI [5]. For the small proportion of BWS with a concomitant paternally transmitted K_ATP_ mutation, their HI were remarkedly severe and prolonged [5]. Somatic features of BWS may not be readily apparent in these patients compared to classical BWS [5].

Herein, we report a case of a large-for-gestational-age infant with medically refractory HI due to a paternally transmitted K_ATP_ mutation, who was subsequently diagnosed with mosaic BWS related to mosaic segmental pUPD (paternal uniparental disomy) 11 based on molecular testing of the pancreatic lesion.

## Case presentation

**Fig. 1:**
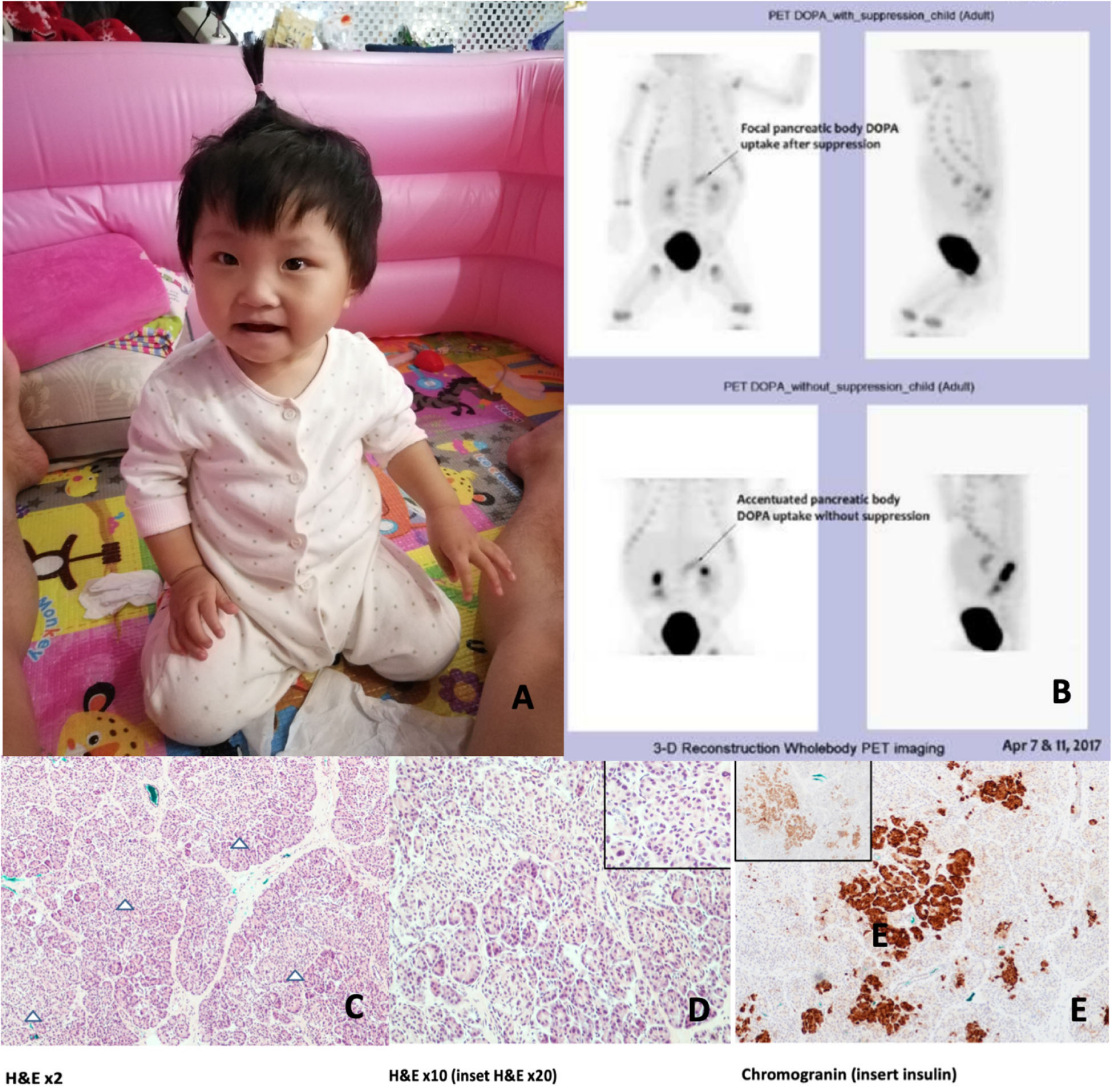
**a**. Picture of the proband with no somatic features suggestive of BWS. **1b.** 18F-Dopa PET scan showed accentuated 18F-dopa uptake in the pancreatic body, with a lesser degree of diffused activity in pancreatic head and tail, suggestive of a focal lesion. **1c-e**. Histology of resected pancreatic tissue. **c.** The pancreas shows preserved acinar architecture with prominent islets of Langerhans (arrowhead), consist of coalescing nests and trabeculae of endocrine cells. **d.** High power field showing some islets containing isolated, enlarged, hyperchromatic nuclei, which is over 2 times the size of the nuclei in the adjacent islet cells. **e.** Immunohistochemical stains confirmed the nests and trabeculae of endocrine cells are positive for neuroendocrine marker chromogranin and many of them express insulin by immunohistochemistry

A female infant was born at 37 weeks of gestation to a non-consanguineous Chinese couple, with a birth weight of 4.3 kg (>2SD). Antenatal history was unremarkable with no gestational diabetes, polyhydramnios nor placentomegaly. She presented with a hypoglycaemic seizure in the first hour of life and required a high glucose infusion rate (GIR) of 20 mg/kg/min to maintain normoglycaemia. Physical examination showed macrosomia but no other dysmorphic features (Fig. 1a). Critical samples taken when blood glucose was 2.8 mmol/L on day 2 of life were compatible with hyperinsulinaemic hypoglycaemia (insulin = 33.9mIU/L, blood beta-hydroxybutyrate < 0.5 mmol/L). She was started on the highest dose of diazoxide (15 mg/kg/day) with hydrochlorothiazide with no response. Octreotide (15mcg/kg/day) was therefore added on with partial response, and she still required a GIR of 11 mg/kg/min.

18F-Dopa PET scan at 3 months of age showed accentuated 18F-dopa uptake in the pancreatic body compared with a lesser degree of diffuse activity in pancreatic head and tail, suggestive of a focal lesion (Fig. 1b). There was no organomegaly or asymmetric kidneys. The first partial pancreatectomy was performed at 5 months of age. A distal lesion was identified by gross inspection intraoperatively, and a distal resection (~ 5% pancreatectomy) was performed. Post-operatively, a high GIR requirement at 11 mg/kg/min was still required. Histology from resected tissue revealed no evidence of pancreatic adenomatosis. Therefore, a second operation was carried out with real-time frozen section evaluation, resulting in an 80% pancreatectomy. Resected pancreatic tissue revealed multiple discrete areas of adenomatosis interspersed between areas of normal exocrine acini. There were areas of coalescing nests and trabeculae (Fig. 1c) that were negative for p57 staining, suggestive of adenomatous hyperplasia; whilst some areas contained islets with isolated enlarged, hyperchromatic nuclei and exocrine acini at the periphery (Fig. 1d). These enlarged nuclei were positive for p57 staining. Post-operatively, the GIR could be further lowered to 3 mg/kg/min but she was unable to be completely weaned off her dextrose infusion. She was subsequently restarted on diazoxide with no response, and hence changed to octreotide. She finally managed to be weaned off from intravenous dextrose with reasonable fasting tolerance of 9 h at the age of 7 months.

### Molecular analysis

Peripheral blood of proband and both parents, and the resected pancreatic tissue (at two different areas: Pancreas 1A and Pancreas 2A) from the proband were collected for genomic DNA extraction and further molecular genetic analysis.

**Fig. 2:**
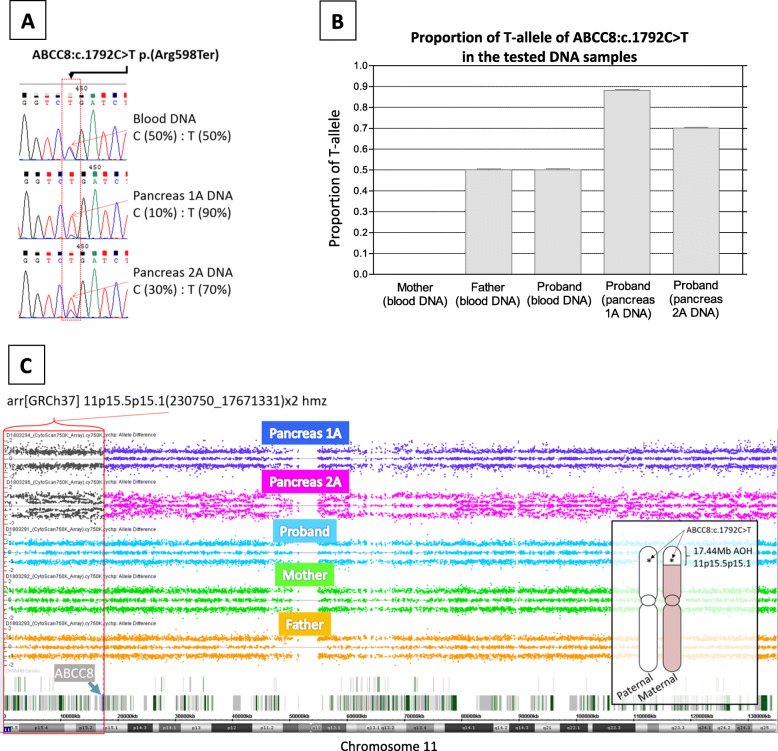
Sanger DNA sequencing and absolute quantitation by digital PCR analyses of ABCC8:c.1792C > T and allele difference plots of chromosome 11. **a** Sanger DNA sequencing results showed C:T allelic ratio in DNA extracted from peripheral blood and Pancreas 1A and 2A tissues of the proband. **b** The proportion of c.1792 T variant in mother, father and proband. The c.1792 T was absent in the mother and presence at 50% in father and proband blood DNA. Pancreas 1A and 2A DNA showed to have 88.8 and 70.6%. of c.1792 T. The proportion of c.1792 T in each DNA sample was averaged of 3 independent assays. Error bars: standard error mean. **c** Allele difference plots of chromosome 11 on DNA extracted from Pancreas 1A and 2A and from peripheral blood of proband and proband’s parents. Red horizontal curly bracket and red vertical rectangle indicate the 17.44 Mb of AOH region in 11p15.5p15.1, arr [GRCh37] 11p15.5p15.1(230750_17671331)× 2 hmz, in Pancreas 1A and 2A. The affected SNPs in Pancreas 1A and 2A are shown in dark grey dots. High level of mosaicism is observed in Pancreas 2A. Vertical dotted line and arrow indicates the location of ABCC8 gene (chr11:17414431–17,498,392, human genome assembly: GRCh37) which is situated with in the AOH region (within red vertical rectangle). Inset (lower right) illustrates the segmental paternal UPD 11p and the location of c.1792C > T in the pancreas DNA, where the beige and white color shaded regions represents maternal and paternal inherited chromosome respectively

A heterozygous pathogenic *ABCC8* NM_000352.4:c.1792C > T p.(Arg598Ter) was found in the DNA extracted from peripheral blood of proband through NGS gene panel analysis. This nonsense loss-of-function c.1792 T variant was previously reported [7]. Sanger DNA sequencing confirmed the NGS finding and confirmed the variant was inherited from the father. The proportion of the c.1792C and c.1792 T variant present in Pancreas 1A and 2A were of approximately 10%:90 and 30%:70%, respectively, estimated by peak height for the c.1792C and c.1792 T in the sequence chromatogram (Fig. 2a). Absolute quantitation by digital PCR analysis showed that the proportion of c.1792 T was approximately 88.9 and 70.6% in Pancreas 1A and 2A, respectively (Fig. 2b), which was concomitant to the level of mosaicism of pUPD11p region carrying the c.1792 T variant inherited from the father.

DNA methylation analysis for chromosome 11p15 showed normal methylation pattern at both IC1 (*H19-IGF2* imprinting centre) and ICR2 (*KCNQ1OT1/KCNQ1* imprinting centre) in the peripheral blood leucocytes of the proband. However, gain of methylation at IC1 and loss of methylation at IC2 were detected in the DNA extracted from resected pancreas, consistent with a diagnosis of Beckwith-Wiedemann Syndrome due to pUPD.

SNP-based chromosomal microarray (CMA) analysis showed copy number neutrality in DNA extracted from the peripheral blood of proband and parents. However, the pancreatic tissue showed a 17.44 Mb region of copy number neutral loss of heterozygosity (LOH) in 11p15.5-p15.1, suggesting segmental UPD 11, with a higher level of mosaicism for Pancreas 1A (~ 90%) compared to Pancreas 2A (~ 70%) (Fig. 2c) for the same region. Trio genotyping analysis on Pancreas 1A and parents using UPDtool [8] based on the SNP genotype results from CytoScan 750 k SNP array further revealed that the 17.44 Mb of LOH in Pancreas 1A was paternally inherited and the rest of the chromosome 11 was biparental, confirming mosaic segmental pUPD 11p15.5-p15.1. CMA was suggested to be a sensitive tool to investigate low level of mosaic segmental UPD [4], however that sensitivity varies between array types in the case of the Affymatrix CytoScan 750 k SNP array, mosaicism above 20% can be detected. Therefore, short tandem repeat (STR) markers analysis as a different molecular approach was performed to verify the level of mosaicism. Based on the peak height ratios of the maternal and paternal alleles detected in the in DNA extracted from Pancreas 1A and 2A (Table 1), paternal allele from D11S1363 to D11S1923 in the 11p15.5-p15.4 region accounted for 83–90% and 68–76% respectively. The results were consistent with the mosaic level of pUPD in 11p15.5-q15.4 shown in CMA. The rest of the chromosome 11 was biparental in both pancreatic sites.

**Table 1:** Analysis of short tandem repeat (STR) marker inheritance and their allelic ratio in pancreas DNA from two different loci (Pancreas 1A and 2A) from the proband

STR	Cytoband	Maternal	Paternal	Pancreas 1A	Pancreas 2A	Pancreas 1A mat:pat allelic ratio	Pancreas 2A mat:pat allelic ratio	Interpretation
D11S1363	11p15.5	a,b	a	a,b	a,b	0.1:0.9	0.24:0.76	mos paternal UPD
D11S1984	11p15.5	a	a,b	a,b	a,b	0.11:0.89	0.25:0.75	mos paternal UPD
CHR11-TH	11p15.5	a,b	c,d	a,c	a,c	0.17:0.83	0.32:0.68	mos paternal UPD
D11S1923	11p15.4	a,b	c,d	b,c	b,c	0.12:0.88	0.26:0.74	mos paternal UPD
D11S1338	11p15.4	a	a,b	a	a	nil	nil	uninformative
D11S904	11p14.2	a,b	c,d	a,d	a,d	1:1	1:1	biparental
D11S2632	11q12	a,b	c	b,c	b,c	1:1	1:1	biparental
D11S956	11q12.1	a,b	c,d	b,d	b,d	1:1	1:1	biparental
D11S898	11q22.1	a	a,b	a,b	a,b	1:1	1:1	uninformative
D11S1299	11q23.3	a,b	a,c	b,c	b,c	1:1	1:1	biparental
D11S488	11q24.1	a,b	c,d	a,d	a,d	1:1	1:1	biparental

mat:pat allelic ratio: maternal to paternal allelic ratio

nil: unable to provide allelic ratio as monoallelic pattern in observed in Pancreas 1A and 2A DNA

biparental: Pancreas 1A and 2A DNA show inheritance of one allele from each parent

mos paternal UPD: the inheritance of alleles in the pancreas 1A and 2A DNA is not unambiguously biparental (due to presence of low level of maternal allele), but is consistent with mosaic UPD of paternal origin

uninformative: unable to delineate inheritance by the STR marker pattern

## Discussion

We described an infant with severe HI resulting from a paternally-inherited *ABCC8* mutation in conjunction with mosaic segmental pUPD11p15 demonstrated in the pancreatic tissue from the second resection but not in peripheral blood leucocytes, suggestive of BWS/BW-spectrum HI. With pUPD11p15, the loss of maternal allele resulted in a loss of *H19* and *CDKN1C* expression, which usually negatively regulates cell proliferation; whereas the biallelic *IGF-II* expression promotes cell growth [9]. Therefore, pancreatic adenomatous hyperplasia and hyperinsulinism were attributed to the combination of the K_ATP_ defect along with the pUPD11 and the imbalance of imprinted genes at 11p15 region. In contrast to the classical histological findings in focal HI related to a paternally-inherited *ABCC8* mutation with lesion confines to a small localized area, our patient had multiple foci of adenomatous hyperplasia throughout the pancreas. Furthermore, the level of mosaicism of UPD cells in the pancreas correlated with the shifted allele frequency of the *ABCC8* mutation. To our understanding, this is the first report using the accurate and sensitive assays to demonstrate the direct correlation of the paternally inherited ABCC8 c.1792 T level with mosaic level of pUPD.

Other than being macrosomic, our patient had no other somatic features of BWS. The consideration of testing for BWS was triggered by the atypical histological findings. This distinct pancreatic histology had been described in children with Beckwith-Wiedemann Spectrum [5, 10, 11]. In a large series of 28 patients with BWS and persistent HI, their phenotypes were reported to range from isolated, subtle hemihypertrophy or umbilical hernia to frank BWS phenotype with multiple somatic features [6]. Only four of them had concomitant K_ATP_ mutations. Therefore, It was suggested that, even in the absence of somatic features of BWS, testing should be considered in HI cases with large ‘focal’ pancreatic lesions with or without a K_ATP_ mutation [5]. The diagnosis of BWS is important due to their inherent vulnerability to embryonal tumours, affecting up to 8% of BWS patients [4, 12]. Calton et al. reported a similar case of large/multifocal focal HI resulting from a paternally inherited recessive *ABCC8* mutation [11]. That patient, like our patient, had no clinical features of BWS. BWS testing was only performed at the age of 20 months when he developed hepatoblastoma. Again, similar to our patient, pUPD11p was identified in the affected tissue (hepatoblastoma tissue and the stored pancreatic tissue), but not in peripheral blood or buccal DNA [11]. This highlights that infants with HI related to mosaic BWS could also develop BWS-associated tumours due to mosaic UPD, and that tumour surveillance is indicated. It has been suggested that the tumour risk could be associated with the level of mosaicism for UPD within specific organs [13]. Since tissues from other organs were not available for testing in our patient, it is unclear whether other organs are affected by pUPD11p. Therefore, tumour surveillance during early childhood is warranted.

With the variability of mosaicism between tissues in patients with BWS, the source of DNA for molecular analysis is extremely important. In our patient, absence of mosaicism in the peripheral blood leukocytes would have wrongly concluded as ‘normal study’ if the pancreatic tissues were not sent for further analysis. Therefore, similar to other mosaic conditions, affected tissue should always be sent for further molecular analysis if possible [4].

## Conclusions

This case highlights the importance of integrating the clinical presentation and subsequent clinical course, together with radiological, genetic and histological findings in the definitive diagnosis of this rare yet clinically important entity. In managing HI caused by both pUPD11p and K_ATP_ mutation, the HI course could be severe, and hypoglycaemia might persist despite extensive pancreatectomy, trial of resuming medical treatment should be considered, allowing better glycaemic control.

## Data Availability

Data sharing is not applicable to this article as no datasets were generated or analysed during the current study.

## Abbreviations

BWS: Beckwith–Wiedemann syndrome
CMA: Chromosomal microarray
GIR: Glucose infusion rate
HI: Hyperinsulinism
LOH: Loss of heterozygosity
UPD: Uniparental disomy
